# Rapid maxillary expansion and its impact on sleep apnea in children aged 5 to 8 years: a retrospective study

**DOI:** 10.1590/2177-6709.30.5.e2524280.oar

**Published:** 2025-12-01

**Authors:** Sarvenaz Zaree BAVANI, Enoch T. NG, Manuel O Lagravère VICH

**Affiliations:** 1 University of Alberta, Faculty of Medicine and Dentistry, Mike Petryk School of Dentistry (Edmon, Canada).

**Keywords:** Rapid maxillary expansion, Apnea-Hypopnea Index, Obstructive sleep apnea, Expansão rápida da maxila, Apneia obstrutiva do sono, Índice de apneia-hipopneia

## Abstract

**Objective:**

Determine the impact of early transverse maxillary expansion on the symptomatic manifestation of sleep apnea in children aged 5-8 years.

**Methods:**

Nineteen patients (mean age: 6.84 years) with maxillary constriction underwent rapid maxillary expansion (RME) for orthodontic treatment. Inclusion criteria: age 5-8 years, Apnea-Hypopnea Index (AHI) > 1, treated with Memory screw Hyrax appliance, under pediatric sleep physician care. Exclusion criteria: non-compliance, re-evaluation delays (> 3 months). Primary variable: AHI before (T0) and after (T1) expansion. Treatment ended based on malocclusion correction. AHI comparisons included dentition status and gender as secondary variables.

**Results:**

A paired t-test showed no significant difference in the mean AHI between T0 (4.05±2.55) and T1 (3.68±3.12) for primary variables (p>0.05). At T0, no significant AHI difference was found between genders (F: 3.21±2.47; M: 4.09±2.87, p>0.05). After expansion (T1), a significant change was observed between genders (F: 1.75±1.03; M: 5.18±3.68, p<0.05). There was also no significant difference in AHI changes based on dentition status. A negative correlation was found between AHI changes and transverse changes and at canine and molar levels.

**Conclusion:**

No significant evidence was found to support the effectiveness of early maxillary expansion in improving sleep apnea among children with maxillary constriction. However, the gender-specific responses and the potential dose-response relationship between maxillary expansion and AHI reduction highlight the complexity of treating pediatric OSA.

## RESUMO

**Objetivo:**

Determinar o impacto da expansão transversal precoce da maxila na manifestação sintomática da apneia do sono em crianças de 5 a 8 anos.

**Métodos:**

Dezenove pacientes (idade média: 6,84 anos) com constrição maxilar foram submetidos a expansão rápida da maxila (EMR) para tratamento ortodôntico. Critérios de inclusão: idade entre 5 e 8 anos, Índice de Apneia-Hipopneia (IAH) > 1, tratamento com aparelho Hyrax com parafuso de memória, sob cuidados de médico pediatra especialista em sono. Critérios de exclusão: não adesão ao tratamento, atrasos na reavaliação (> 3 meses). Variável primária: IAH antes (T0) e após (T1) a expansão. O tratamento foi encerrado com base na correção da má oclusão. As comparações do IAH incluíram o estado da dentição e o sexo como variáveis secundárias.

**Resultados:**

O teste t pareado não mostrou diferença significativa na média do IAH entre T0 (4,05±2,55) e T1 (3,68±3,12) para as variáveis primárias (p>0,05). Em T0, não foi encontrada diferença significativa no IAH entre os sexos (F: 3,21 ± 2,47; M: 4,09 ± 2,87, p>0,05). Após a expansão (T1), observou-se uma mudança significativa entre os sexos (F: 1,75±1,03; M: 5,18±3,68, p<0,05). Também não houve diferença significativa nas mudanças do IAH com base no status da dentição. Foi encontrada uma correlação negativa entre as mudanças do IAH e as mudanças transversais e nos níveis dos caninos e molares.

**Conclusão:**

Não foram encontradas evidências significativas que comprovem a eficácia da expansão maxilar precoce na melhoria da apneia do sono em crianças com constrição maxilar. No entanto, as respostas específicas de cada sexo e a potencial relação dose-resposta entre a expansão maxilar e a redução do IAH destacam a complexidade do tratamento da AOS pediátrica.

## INTRODUCTION

Obstructive sleep apnea (OSA) is the most prevalent sleep-related breathing disorder, characterized by temporary cessations in breathing (apnea) or significant reductions in breathing amplitude (hypopnea) due to an obstructed or collapsed upper airway. These events result in decreased blood oxygen levels (hypoxemia) and increased blood carbon dioxide levels (hypercapnia).¹^,^² The severity of OSA is commonly quantified using the apnea-hypopnea index (AHI), which describes the total number of apnea and hypopnea episodes per hour of sleep (Table 1).³

**Table 1:** Apnea/hypopnea index ( AHI ) in children.

AHI score	Severity
<1	Normal
1 - 4.9	Mild
5 - 9.9	Moderate
>9	Severe

The relationship between sleep apnea and maxillary constriction has been studied in the context of pediatric OSA.⁴^-^⁶ Research has shown that maxillary constriction is thought to increase nasal resistance and alter tongue position, leading to the narrowing of the retroglossal airway, which is a characteristic feature of OSA.⁴ Additionally, increased upper airway resistance, including narrowing or retro-positioning of the maxilla or mandible, is correlated with the severity of OSA in children.⁵ Furthermore, studies have highlighted the association between maxillary constriction and oropharyngeal space reduction, nasal resistance increase, tongue retro-position, and upper airway narrowing, all of which are relevant to the pathophysiology of pediatric OSA.⁶ These findings support the link between maxillary constriction and the pathogenesis of pediatric OSA.

Several studies have explored the impact of maxillary expansion on the craniofacial and nasal areas in children, as well as its potential to alleviate OSA symptoms.⁷^-^¹⁰ A systematic review and meta-analysis concluded that palatal expansion in pediatric patients under the age of 18 decreases nasal resistance and increases nasal flow, indicating a positive impact on nasal breathing.⁷ Additionally, rapid maxillary expansion (RME) has been shown to lead to separation of the midpalatal suture, improving the occlusion and modifying craniofacial growth, which may have implications for the management of conditions such as OSA in children.⁸^,^⁹ Furthermore, an umbrella review reported significant and stable increases in the nasal and oropharyngeal space volumes, as well as a decrease in airway resistance in children and adolescents under the age of 18 who underwent RME.¹⁰

While some studies have suggested that RME is effective in improving OSA in children, particularly in terms of the AHI and oxygen saturation,⁹^-^¹⁰ further investigation is warranted, especially in younger pediatric patients. Therefore, this research aimed to address this knowledge gap by specifically examining the impact of RME on OSA in children younger than eight years. The study focused on children with a narrow maxilla who were undergoing multidisciplinary treatment for OSA, with the objective of evaluating the effect of maxillary expansion on OSA without interrupting their ongoing treatment.

## MATERIAL AND METHODS

This study was approved by Alberta Research Information Services: Human Research Ethics Board (Pro00142659). The study followed a retrospective design. Records taken from patients treated between October 2021 and August 2023 were reviewed. Patients with maxillary constriction who underwent RME as part of their orthodontic treatment at a general private practice were selected. The final sample comprised 19 pediatric patients, with a mean age of 6.84 years, ranging from 5 to 8 years. Of these, 8 were females and 11, males. Records were anonymized to safeguard patient privacy and confidentiality.

The main objective of the study was to determine potential improvements in OSA by means of reducing the AHI among patients who underwent palatal expansion as a corrective measure for maxillary constriction. However, it should be noted that the expansion procedure was fundamentally driven by dental and skeletal considerations, with the potential impact on OSA serving as a secondary consideration in the decision to commence treatment at a younger age. Additionally, the patients, while undergoing palatal expansion, were scheduled to receive specialized sleep treatment by sleep specialists.

It is important to note that in our jurisdiction, the evaluation and diagnosis of pediatric OSA fall outside the scope of practice for dentists. Due to the nature of wait lists and wait times for pediatric sleep physician specialists in our area, sleep screening and sleep testing are provided to patients. Once results are reviewed by an appropriate physician, referral is then made for the patients to pediatric sleep medicine. Current wait times for a polysomnography (PSG) are approximately three years, and wait times with sleep study results for pediatric pulmonology are approximately one year. For this reason, adjunctive orthodontics is provided in patients where orthodontic expansion is indicated to solve skeletal transverse deficiency and where the diagnosing physician has recommended adjunctive dental treatment while the patient is still on the waitlist for a pediatric sleep specialist for initial evaluation.

The database was filtered using the following criteria:

1. Presence of malocclusion characterized by transverse maxillary deficiency such that RME would be an appropriate treatment based on orthodontic findings.
2. Participants were children eight years old and younger at the time of expansion.
3. Participants had a diagnosis of OSA (AHI > 1) as defined by a home sleep test.
4. Participants were treated with the RME appliance (Memory Screw Hyrax).
5. Pre- and post-treatment AHI scores were at least 3 months apart.

Exclusion criteria:

1. Any participant who did not cooperate with the treatment.
2. Patients who were extremely delayed (greater than 3 months) in returning for re-evaluation.

## STUDY DESIGN

It is important to emphasize that the primary purpose of maxillary expansion in this study was to address maxillary constriction, which is an orthodontic indication. While the potential impact on OSA was considered, it was not the primary goal of the treatment. The AHI measurements were used as a secondary outcome to assess any potential benefits of the orthodontic treatment on sleep-related breathing disorders.

Comprehensive personal and family histories from all participants were gathered, and thorough clinical examination was conducted. Additionally, each child underwent an orthodontic assessment to identify potential deviations from normal occlusion. A RME device was applied in all the children who met the inclusion criteria of constricted maxilla. These patients also underwent testing for OSA with a home sleep test to determine their AHI before the application of the RME appliance (T0). Any patient with AHI >1 was referred to pediatric pulmonology/sleep medicine for in-person assessment and medical evaluation and treatment for pediatric OSA.

**Figure 1:**
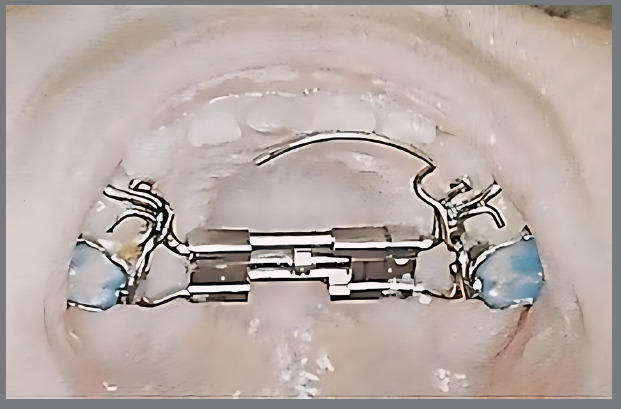
RME appliance (Memory Screw Hyrax).

The appliance used was a fixed two-band RME appliance (Memory Screw Hyrax) with a conventional expansion screw along with an integrated spring, to provide a constant activation level over the treatment period. The Memory Screw Hyrax was fitted to the maxillary second deciduous molars with an occlusal rest on the first deciduous molars (Fig. 1). Expansion was achieved by activating an axial screw (0.25mm per turn, full rotation of 1mm at 800g of force). Parents were instructed to activate the screw four times per week, with the flexibility of activating the screw four times in a single day instead of equally spacing the activation throughout the week. The treatment phase ended upon the dentist’s determination that further expansion was not advisable for malocclusion correction. Subsequently, the RME device was replaced with an upper holding arch appliance, to maintain the achieved maxillary expansion until re-evaluation. Using a home sleep test, reevaluation (T1) was conducted for OSA to see if there was any decrease in the AHI value.

## STATISTICAL ANALYSIS

In this study, data were expressed as mean ± SD (standard deviation). The reliability and significance of the data were assessed using a paired t-test to compare the AHI scores before and after maxillary expansion.

The measurements were taken at two time points: before the application of the RME appliance (T0) and after a period of at least three months following the expansion (T1). Statistical significance was set at a p-value <0.05.

## RESULTS

The study was conducted from October 2021 to August 2023, involving a cohort of 19 participants (11 males and 8 females) with an age range of 5 to 8 years and a mean age of 6.84 years (SD = 1.6). Thirteen participants were in the mixed dentition stage, and six were in the primary dentition stage. The pre-expansion and post-expansion AHI measurements were compared using paired t-tests, which are appropriate for assessing differences between two related groups. A significance level was set at p < 0.05 to determine statistical significance.

The analysis revealed that the mean pre-expansion AHI was 4.05 ± 2.55, and the mean post-expansion AHI was 3.84 ± 3.21. There was no statistically significant difference between the pre-expansion and post-expansion AHI measurements, with a p-value of 0.94. This suggests that the observed variations in AHI values may be attributed to natural variability rather than a significant treatment effect of maxillary arch expansion. Prior to the expansion, there was no statistically significant difference in AHI between male and female participants (p >0.05). However, following the expansion, a significant difference in AHI between male and female participants was observed (p < 0.05) (Table 2).

**Table 2:** Gender variability and its impact on AHI changes before and after maxillary arch expansion.

	Gender		p-value
	Male	Female	
Mean AHI before maxillary expansion	4.09 ± 2.87	3.21 ± 2.47	0.44
Mean AHI after maxillary expansion	5.18 ± 3.68	1.75 ± 1.03	0.01*
Mean of magnitude AHI Changes (± SD)	1.09 ± 3.6	-1.37 ± 2.55	0.1

(p-value* = statistically significant).

**Figure 2:**
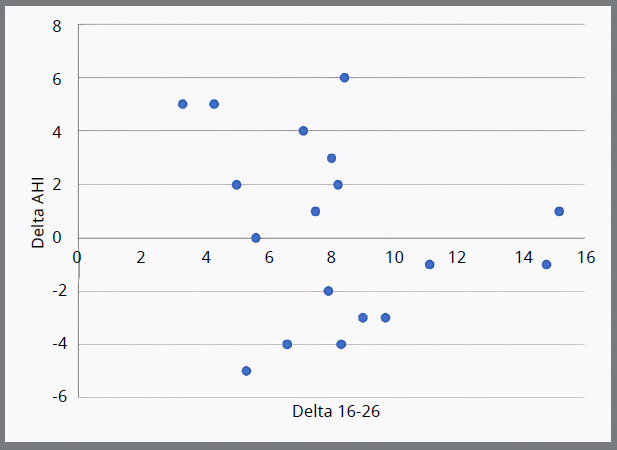
Changes in transverse dimensions at the canine and first molar levels (Delta CC and Delta 16-26) as well as the change in the Apnea-Hypopnea Index (Delta AHI) following an intervention.

Furthermore, no statistically significant difference in the magnitude of AHI changes between the primary and mixed dentition groups was found (p > 0.05) (Table 3). The transverse change at the primary canine and primary second molar (in cases where the first molar had not erupted in the arch) showed a mean expansion of 8.47 ± 2.3 and 7.9 ± 3.1, respectively. The mean difference in AHI (Delta AHI) was 0.10 ± 3.4, and a negative correlation was found between the transverse change and AHI at both canine and molar levels (-0.24 and -0.18) (Table 4, Fig. 2).

**Table 3:** Difference in magnitude of AHI changes when the expansion performed as early as in the primary dentition stage, as compared to expansion performed slightly later in the mixed dentition stage.

	Dentition	
	Primary	Mixed
Mean (± SD) of magnitude of AHI changes	-1.5 ± 2.5	0.76 ± 3.5
p-value		0.14

**Table 4:** Summary of the mean and standard deviation of the changes in transverse dimensions at the canine and molar level as well as the change in the Apnea-Hypopnea Index following an intervention and correlation between expansion and AHI.

Expansion level	Mean expansion ± SD	Mean AHI before expansion ± SD	Mean AHI after expansion ± SD	Correlation between expansion and AHI
Canine to canine	8.47 + 2.34	4.05 ± 2.55	3.84 ± 3.21	-0.24
Molar to molar	7.99 + 3.11			-0.18

## DISCUSSION

This study aimed to explore the impact of maxillary arch expansion on the AHI in young children diagnosed with maxillary transverse deficiency and OSA. The primary focus was on addressing maxillary constriction while also assessing the intervention’s impact on managing OSA. This focus on very young children, a population not extensively studied, provides a unique perspective on the effects of early maxillary expansion. The results revealed no statistically significant change in AHI following maxillary expansion, with a p-value ≥ 0.05. 

This contrasts with existing literature suggesting that RME can effectively reduce AHI in children with OSA.^11^ Several factors may contribute to this disparity. Firstly, the variability in AHI measurements and the natural variability in AHI could obscure the treatment effect.^11^ Secondly, the small sample size and specific age range of our cohort might limit the generalizability of our findings and their comparability with broader studies. Thirdly, the heterogeneity in the severity of sleep apnea among participants could have influenced the effectiveness of maxillary expansion. Notably, a significant decrease in AHI was observed for female participants following expansion, while male participants showed an increase. This gender-based difference aligns with existing literature indicating variations in the pathophysiology and treatment response of OSA between genders.^12^ Factors such as differences in upper airway anatomy, fat distribution, and hormonal influences might modulate the gender differences in OSA and its response to treatments like maxillary expansion.^12^

Additionally, the timing of puberty, which differs between males and females, could play a role in these disparate outcomes. Girls typically begin puberty earlier than boys, which may influence craniofacial growth patterns and airway development.^13^ This developmental difference could potentially explain the observed decrease in AHI for females and increase for males in our study. The analysis did not identify a statistically significant difference in AHI changes between the primary and mixed dentition stages, suggesting that the timing of maxillary expansion in relation to the dentition stage might not critically impact its effectiveness in reducing AHI. This observation deserves further investigation, as the literature lacks consensus on the optimal timing for maxillary expansion to achieve the best outcomes in managing pediatric sleep apnea.^10^ However, it is important to note that early treatment with RME has been shown to produce significant favorable postpubertal modifications in both maxillary and mandibular structures, whereas late treatment induced only a significant restriction of mandibular growth.^13^

The study revealed a trend where greater maxillary expansion was associated with a more substantial reduction in the AHI, despite the lack of statistical significance. This observation suggests a potential dose-response relationship between maxillary expansion and AHI reduction, underscoring the need for further investigation into this association. These results are consistent with prior research, which has indicated that AHI reduction in pediatric OSA patients undergoing maxillary skeletal expansion is linked to upper airway enlargement rather than nasomaxillary complex expansion.^14^ This emphasizes the importance of comprehensive approaches that consider factors beyond skeletal modification alone. The multifactorial nature of pediatric OSA contributes to the heterogeneous treatment responses observed in children, highlighting the nuanced nature of therapeutic interventions such as maxillary expansion.^6^ ^,^ ^10^ Individual anatomical variations, including the size of tonsils and adenoids, craniofacial morphology, and the presence of comorbidities, significantly influence the efficacy of OSA treatments in pediatric patients.^15^ ^-^ ^17^

Therefore, while maxillary expansion may offer benefits for certain pediatric patients, its effectiveness is contingent upon a myriad of interconnected anatomical and physiological factors,^10^ ^,^ ^11^ emphasizing the need for tailored and multidisciplinary approaches to pediatric OSA treatment.^6^ ^,^ ^18^ In the context of craniofacial morphology and its relationship with OSA, it is noteworthy that the complex interplay between OSA and Class II malocclusion, along with specific craniofacial features, is multifaceted. While certain craniofacial characteristics, such as mandibular retrognathia commonly seen in Class II malocclusions, are associated with a higher risk of OSA due to a narrower upper airway, the evidence does not conclusively establish a direct causal link. Further research with robust methodological designs is needed to clarify the extent to which these craniofacial features contribute to the development and severity of OSA in children.^19^

It should be noted that all these patients did start with an official diagnosis of OSA. Several factors may have influenced the results of this study, which should be considered when interpreting the findings. The first limitation is the relatively small sample size, which may limit the statistical power and the generalizability of the results. An additional limitation that deserves discussion is the lack of multi-night testing for the AHI. Single-night AHI measurements may not accurately represent the severity of OSA, due to night-to-night variability in AHI values. This variability can lead to misclassification of disease severity, potentially affecting the interpretation of the relationship between maxillary expansion and AHI reduction. Multi-night testing could provide a more accurate assessment of OSA severity and the impact of maxillary expansion on AHI.^20^ In addition to these limitations, our research did not account for adenoid and tonsillar enlargement, nor did we exclude patients with adenoid and tonsillar enlargement from the study. 

The presence of enlarged tonsils and adenoids is a known contributing factor to OSA in children, as they can significantly obstruct the airway.^21^ By not excluding these patients, the study may have included a subset of participants for whom maxillary expansion might not be as effective, due to these additional obstructions. Furthermore, the study did not formally assess nasal patency or dimensions, which are critical factors in the pathophysiology of sleep apnea. Nasal obstruction can contribute significantly to the development and severity of OSA by increasing upper airway resistance and altering breathing patterns during sleep.^22^ The lack of assessment for nasal patency means that the study may not have fully captured the multifactorial nature of sleep apnea, nor could it accurately determine the extent to which maxillary expansion might alleviate sleep apnea symptoms by improving nasal airflow. It is important to acknowledge the inherent limitations of the retrospective design employed in this study. 

Retrospective studies, while valuable for generating hypotheses and examining existing data, are subject to certain constraints. These include potential selection bias, as the data was not collected specifically for this research question. There may also be incomplete or inconsistent data, as retrospective studies rely on previously recorded information. Additionally, the lack of randomization and control groups in retrospective designs limits our ability to establish causality definitively. These factors may affect the generalizability of our findings and should be considered when interpreting the results. Based on the findings and limitations of this study, future research should consider several key improvements to enhance our understanding of maxillary expansion’s role in managing pediatric OSA. Firstly, conducting prospective, controlled studies with larger sample sizes would address the limitations of the current retrospective design and small cohort, enhancing statistical power and generalizability. Secondly, implementing multi-night sleep studies could provide more accurate AHI measurements, reducing the impact of night-to-night variability and improving the assessment of OSA severity and treatment outcomes. Lastly, incorporating comprehensive assessments of anatomical factors, including formal evaluation of nasal patency, adenoid and tonsillar size, and craniofacial morphology, would offer a more nuanced understanding of the relationship between maxillary expansion and OSA. 

This multifaceted approach would account for the complex interplay of factors influencing treatment efficacy in pediatric patients, potentially leading to more tailored and effective treatment protocols. By addressing these aspects, future studies could significantly contribute to the evidence base and guide clinical decision-making in the management of pediatric OSA. The implications of these findings for clinical practice are significant. While existing literature generally supports maxillary expansion as a non-invasive intervention for pediatric OSA,^10^ ^,^ ^23^ ^,^ ^24^ the results of this study emphasizes the importance of critically evaluating the evidence base and tailoring treatment strategies to individual patient profiles. The development of personalized approaches that account for patient-specific characteristics, including sex differences, pubertal stage, and craniofacial growth patterns, is essential for optimizing therapeutic outcomes in pediatric OSA management.^18^ ^,^ ^13^ ^,^ ^25^ ^,^ ^26^

## CONCLUSION

In conclusion, although this study did not find significant evidence supporting the effectiveness of early maxillary expansion in improving sleep apnea among children with maxillary constriction, the gender-specific responses and the potential dose-response relationship between maxillary expansion and AHI reduction highlight the complexity of treating pediatric OSA. These findings underscore the importance of developing personalized treatment strategies and emphasize the need for further research to enhance therapeutic outcomes in pediatric OSA management.
